# Prognostic factors of T2aN0M0 (T3‐4cmN0M0, stage IB) non‐small‐cell lung cancer after surgery: Single‐center real‐world research

**DOI:** 10.1111/1759-7714.14204

**Published:** 2021-11-03

**Authors:** Lei Liu, Jiaqi Zhang, Guige Wang, Ke Zhao, Chao Guo, Yeye Chen, Cheng Huang, Shanqing Li

**Affiliations:** ^1^ Department of Thoracic Surgery Peking Union Medical College Hospital, Chinese Academy of Medical Sciences Beijing China

**Keywords:** IB stage, non‐small‐lung cancer, prognosis, surgery

## Abstract

**Aim:**

To further elucidate the prognostic factors of non‐small‐cell lung cancer (NSCLC) patients with T2aN0M0 (stage IB) who underwent surgical treatment.

**Methods:**

We retrospectively analyzed the data of stage IB NSCLC patients who underwent surgical treatment at our center from October 2013 to September 2016. Eighty patients were enrolled. We analyzed their overall survival (OS) and disease‐free survival (DFS) using the Kaplan–Meier method.

**Results:**

In univariable analysis, adenosquamous carcinoma (ASC) was significantly associated with inferior DFS (*p* = 0.036, *p* = 0.037) and OS (*p* = 0.001, *p* = 0.003) in all stage IB patients and those who only accepted surgery. Patients with a number of N2 lymph node dissections of ≥3 regions (N2‐LSNDr) exhibited better DFS (*p* = 0.020, *p* = 0.005) and OS (*p* = 0.003, *p* = 0.001) in all stage IB patients and those who only accepted surgery. In addition, advanced age (≥70 years old) is an adverse factor for DFS (*p* = 0.049) and OS (*p* = 0.018) among patients who did not receive adjuvant chemotherapy following surgery. In multivariable analyses, patients with N2‐LSNDr exhibited a longer OS (*p* = 0.045) in all enrolled patients; patients with N2‐LSNDr (*p* = 0.016) and younger age (*p* = 0.021) demonstrated a superior OS in patients who only received surgery.

**Conclusions:**

We found that N2‐LSNDr were independent influencing factors affecting the prognosis in all included stage IB patients and stage IB patients without adjuvant chemotherapy. ASC was associated with worse prognosis of T2aN0M0 NSCLC. Older age is an independent prognostic factor of the worst OS in stage IB patients without adjuvant chemotherapy.

## Graphical abstract

To further elucidate the prognostic factors of non‐small‐cell lung cancer (NSCLC) patients with T2aN0M0 (stage IB) who underwent surgical treatment. We retrospectively analyzed the data of stage IB NSCLC patients who underwent surgical treatment at our center from October 2013 to September 2016 and 80 patients were enrolled. We analyzed their overall survival (OS) and disease‐free survival (DFS) using the Kaplan–Meier method. In univariable analysis, adenosquamous carcinoma (ASC) was significantly associated with inferior DFS (*p* = 0.036, *p* = 0.037) and OS (*p* = 0.001, *p* = 0.003) in all stage IB patients and those who only accepted surgery. Patients with a number of N2 lymph node dissection of ≥3 regions (N2‐LSNDr) exhibited better DFS (*p* = 0.020, *p* = 0.005) and OS (*p* = 0.003, *p* = 0.001) in all stage IB patients and those who only accepted surgery. In addition, advanced age (≥70 years old) is an adverse factor for DFS (*p* = 0.049) and OS (*p* = 0.018) among patients who did not receive adjuvant chemotherapy following surgery. In multivariable analyses, patients with N2‐LSNDr exhibited a longer OS (*p* = 0.045) in all enrolled patients; patients with N2‐LSNDr (*p* = 0.016) and younger age (*p* = 0.021) demonstrated a superior OS in patients who only receive surgery. We found that N2‐LSNDr were independent influencing factors affecting the prognosis in all included stage IB patients and stage IB patients without adjuvant chemotherapy. ASC was associated with worse prognosis of T2aN0M0 NSCLC. Older age is independent prognostic factor of the worse OS in stage IB patients without adjuvant chemotherapy.

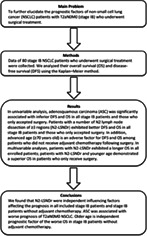

## INTRODUCTION

Lung cancer is the leading cause of malignant tumor‐related deaths worldwide.^1^ With the constant advancement of surgical technology and drug development, lung cancer treatment, particularly non‐small‐cell lung cancer (NSCLC), is undergoing constant changes, including segmentectomy,^2^ targeted therapy,^3^ immunotherapy,^4^ and so on.

According to literature reports, the 5‐year survival rate for patients with stage IA1, IA2, and IA3 is ≥80%, whereas that for patients with stage IB is reduced to 73%.^5^ According to the eighth edition of the TNM tumors nodes and metastases (TNM) staging system, only three conditions are judged as stage IB: T2aN0M0 (T3‐4cmN0M0), T2*Centr*N0M0, and T2*Visc* PIN0M0. According to the latest guidelines, high‐risk stage IB patients should receive adjuvant chemotherapy or targeted therapy with osimertinib. High‐risk factors include poorly differentiated tumors (including lung neuroendocrine tumors [excluding well‐differentiated neuroendocrine tumors]), vascular invasion, wedge resection, tumors >4 cm, visceral pleural involvement, and unknown lymph node status (Nx). However, the guidelines also point out that these factors cannot be independently considered an indication and may be considered when determining adjuvant chemotherapy treatment.^6^ In addition, we have observed in clinical practice that stage IB prognosis is much different from that of stage IA, and the formulation of treatment plans for T2aN0M0 patients is often ambiguous. Therefore, we conducted a retrospective study on stage IB NSCLC patients who underwent surgery at our center, focusing on stage IB patients with tumors of 3–4 cm in diameter, or T2aN0M0. We hoped to determine the factors that influence the prognosis of NSCLC patients at this stage and develop an appropriate treatment plan for these patients.

## METHODS

### Patient population

This study retrospectively analyzed the following parameters for patients who underwent surgical treatment with pathological stage of T2aN0M0 (T3‐4cmN0M0) at our center from October 2013 to September 2016: age, sex, smoking history, surgical methods, pathological subtypes, lymph node dissection, postoperative adjuvant treatment, survival status, tumor metastasis status, and so on. All of the patients in the group were restaged according to eighth edition UICC‐TNM classification. We define people with age ≥70 as the older age group and those with age <70 as the younger age group. This retrospective study was performed under authorization approved by the Institutional Review Board of Peking Union Medical College Hospital, Beijing, China.

### Lymph node dissection

In 2005, the International Association for the Study of Lung Cancer (IASLC) proposed a definition of complete resection for NSCLC.^7^ Systematic nodal dissection (ND) or lobe‐specific nodal dissection (LSND) is widely recommended. In particular, LSND implies dissection and histological examination of intrapulmonary (regions 11 and following) and hilar (region 10) nodes and at least three N2 regions depending on lobar location of the primary tumor. Analysis of regions 7 and 10 is mandatory regardless of tumor location. The minimal number of resected lymph nodes is three from the N1 and N2 regions. N1 denotes lymph nodes contained within the pleural reflection (hilar and parenchymal, stations 10–14); N2 denotes lymph nodes in ipsilateral mediastinal nodes outside the pleural reflection (stations 2–9).^8^ According to the above definition, we define the number of lymph node dissection ≥3 regions as LSNDr, <3 regions as non‐LSNDr, the number of lymph node dissection ≥10 as LSNDn, and <10 as non‐LSND.

### Follow‐up

Routine surveillance after operation completion, including physical examination, blood tests, and chest CT scan, was conducted every 3–6 months for 5 years, whereas bone scan, head‐enhanced MRI, and PET/CT examination were performed every year to rule out distant metastases. Patients were subjected to systemic examinations when symptoms or signs recurred. The duration of overall survival (OS) was defined as the interval between the date of surgical resection and death. The duration of disease‐free survival (DFS) was defined as the interval between the date of surgical resection and locoregional recurrence, distant recurrence or death from any cause.

### Statistical analysis

The SPSS software package, version 25.0 (IBM, SPSS Statistics, Chicago, IL, USA), was utilized for data analysis. A rank‐sum test was employed to analyze the differences in variables between the groups. The Kaplan–Meier method was used to estimate OS and DFS in all patients and subgroups. The log‐rank test was deployed for single‐factor analysis and Cox regression analysis was utilized for multivariate analysis. A multivariate analysis included factors identified in univariate analyses with *p* values <0.05. The multivariate regression analysis included variables with statistical significance in the univariate analysis. A *p* value of <0.05 was considered to be statistically significant.

## RESULTS

### Demographic data

This study enrolled 80 patients, with a male to female ratio of 51:29 and an average age of 48 (range 41–86) years old. The median follow‐up time was 72 (range 53–91) months. Adjuvant chemotherapy was administered to 14 patients following surgery. The main reasons for chemotherapy were poor differentiation (5/14), vascular nerve invasion (1/14), papillary components (5/14), and mucinous adenocarcinoma pathological subtypes (3/14). The chemotherapy regimen was based on platinum (cisplatin or carboplatin), combined with pemetrexed or gemcitabine. None of the patients included in this study received targeted therapy or immunotherapy for first‐line adjuvant therapy after surgery. At the last follow‐up, 14 patients had tumor metastases, with a median DFS of 67 (range 5–91) months, and 15 patients died, with a median OS of 68 (range 6–91) months. Table 1 summarizes the general conditions of enrolled patients.

**TABLE 1:** Characteristics of 80 stage IB NSCLC patients

Variable	*N* (%)
Gender	
Male	51 (63.75)
Female	29 (36.25)
Age (years)	48 (range 41–86)
Younger group	67 (83.75)
Older group	13 (16.25)
Smoking history	
Yes	36 (45.00)
No	44 (55.00)
Adjuvant chemotherapy	
Yes	14 (17.50)
No	66 (82.50)
Pathology subtype	
Adenocarcinoma	47 (58.75)
Squamous cell carcinoma	25 (31.25)
Adenosquamous carcinoma	3 (3.75)
Other^a^	4 (5)
*T*	
≤3.5 cm	47 (58.75)
>3.5 cm	33 (41.25)
N1 lymph nodes dissection	
LSND (number)	
Yes	46 (57.50)
No	34 (42.50)
LSND (region)	
Yes	28 (35.00)
No	52 (65.00)
N2 lymph nodes dissection	
LSND (number)	
Yes	63 (78.75)
No	17 (21.25)
LSND (region)	
Yes	67 (83.75)
No	13 (16.25)
Vascular invasion	
Yes	4 (5.00)
No	76 (95.00)
Surgical procedure	
Lobe dissection	77 (96.25)
Sublobe dissection	3 (3.75)

^a^ Large cell neuroendocrine carcinoma, bronchial carcinosarcoma, carcinoid, malignant melanoma.

### Univariable analysis of prognostic factors

**FIGURE 1:**
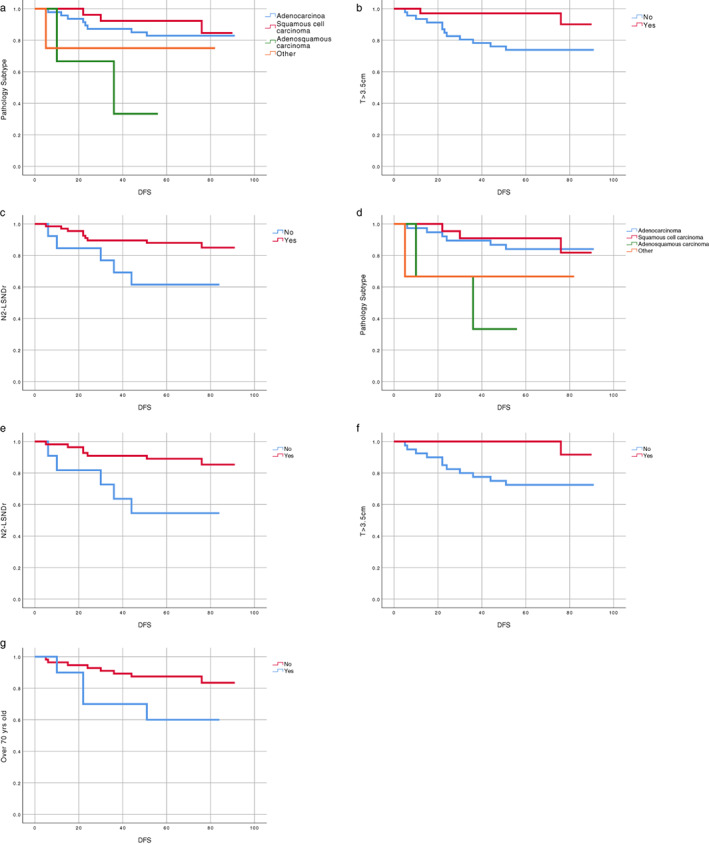
Kaplan‐Meier survival curves of disease‐free survival (DFS) for all the enrolled stage IB patients: a. pathology subtype; b. tumor size; c. LSNDr status. Kaplan‐Meier survival curves of DFS for stage IB patients who only receive surgery: d. pathology subtype; e. LSNDr status; f. tumor size; g. age

In univariable analysis of DFS, among all the enrolled stage IB patients, we found that patients with adenosquamous carcinoma (ASC) had worse DFS (univariable hazard ratio [HR] 0.35, 95% confidence interval [CI] 0.04–3.41, *p* = 0.036), whereas patients with N2‐LSNDr (univariable HR 3.41, 95% CI 1.13–10.23, *p* = 0.020) and those with tumor size (*T*) > 3.5 cm (univariable HR 4.91, 95% CI 1.10–21.93, *p* = 0.021) had longer DFS (Table 2). Among stage IB patients who did not receive adjuvant chemotherapy following surgery, we found that patients with ASC had inferior DFS (univariable HR 0.29, 95% CI 0.03–2.84, *p* = 0.037) through univariate analysis. Patients with N2‐LSNDr (univariable HR 4.45, 95% CI 1.40–14.13, *p* = 0.005) and *T* > 3.5 cm (univariable HR 8.25, 95% CI 1.07–63.97, *p* = 0.016) had better DFS. In addition, advanced age (≥70 years old) is an adverse factor for DFS (univariable HR 0.32, 95% CI 0.10–1.07, *p* = 0.049) (Figure 1 and Table 3).

**TABLE 2:** Univariable and multivariable analyses of disease‐free survival and overall survival for all the enrolled stage IB patients

Variable	Univariable analysis			Multivariable analysis		
	HR	95% CI	*p*	HR	95% CI	*p*
Disease‐free survival						
Gender	1.42	0.49–4.09	0.517			
Age	2.21	0.69–7.06	0.180			
Smoking history	0.86	0.30–2.49	0.783			
Adjuvant Chemotherapy	0.79	0.18–3.54	0.760			
Pathology subtype	0.35	0.04–3.41	0.036*	0.79	0.21–3.00	0.726
*T* > 3.5 cm	4.91	1.10–21.93	0.021*	5.42	1.19–24.68	0.029*
N1 LSND (number)	0.71	0.25–2.04	0.529			
N1 LSND (region)	0.70	0.22–2.22	0.538			
N2 LSND (number)	0.62	0.19–1.98	0.419			
N2 LSND (region)	3.41	1.13–10.23	0.020*	3.48	0.89–13.52	0.072
Surgical procedure	1.94	0.25–14.86	0.525			
Overall survival						
Gender	0.66	0.21–2.07	0.474			
Age	2.77	0.95–8.14	0.063			
Smoking history	1.30	0.47–3.60	0.611			
Adjuvant Chemotherapy	0.34	0.04–2.57	0.294			
Pathology subtype	0.46	0.05–4.14	0.001*	0.87	0.26–2.91	0.824
*T* > 3.5 cm	0.48	0.15–1.51	0.208			
N1 LSND (number)	1.09	0.39–3.07	0.871			
N1 LSND (region)	0.67	0.21–2.10	0.490			
N2 LSND (number)	0.51	0.17–1.50	0.223			
N2 LSND (region)	4.31	1.53–12.12	0.003*	3.41	1.03–11.45	0.045*
Surgical procedure	1.42	0.19–10.83	0.737			

Abbreviations: CI, confidence interval; HR, hazard ratio, LSND, lobe‐specific nodal dissection.

* Statistically significant.

**TABLE 3:** Univariable and multivariable analyses of disease‐free survival and overall survival for stage IB patients only receive surgery

Variable	Univariable analysis			Multivariable analysis		
	HR	95% CI	*p*	HR	95% CI	*p*
Disease‐free survival						
Gender	0.96	0.29–3.19	0.946			
Age	0.32	0.10–1.07	0.049*	0.45	0.11–1.79	0.255
Smoking history	1.02	0.33–3.16	0.974			
Pathology subtype	0.29	0.03–2.84	0.037*	0.49	0.04–5.51	0.563
*T* > 3.5 cm	8.25	1.07–63.97	0.016*	7.96	1.00–63.51	0.050
N1 LSND (number)	0.66	0.21–2.06	0.478			
N1 LSND (region)	0.62	0.17–2.29	0.473			
N2 LSND (number)	0.75	0.20–2.78	0.666			
N2 LSND (region)	4.45	1.40–14.13	0.005*	4.00	0.92–17.45	0.065
Surgical procedure	1.90	0.24–14.76	0.541			
Overall survival						
Gender	0.51	0.14–1.81	0.297			
Age	0.29	0.10–0.87	0.018*	0.24	0.07–0.81	0.021*
Smoking history	1.28	0.44–3.69	0.653			
Pathology subtype	0.42	0.05–3.75	0.003*	0.78	0.08–7.45	0.826
*T* > 3.5 cm	0.38	0.11–1.38	0.143			
N1 LSND (number)	1.27	0.43–3.81	0.665			
N1 LSND (region)	0.80	0.25–2.55	0.704			
N2 LSND (number)	0.45	0.15–1.36	0.159			
N2 LSND (region)	4.88	1.69–14.10	0.001*	4.69	1.33–16.54	0.016*
Surgical procedure	1.23	0.16–9.47	0.841			

Abbreviations: CI, confidence interval; HR, hazard ratio; LSND, lobe‐specific nodal dissection. *Statistically significant.

**FIGURE 2:**
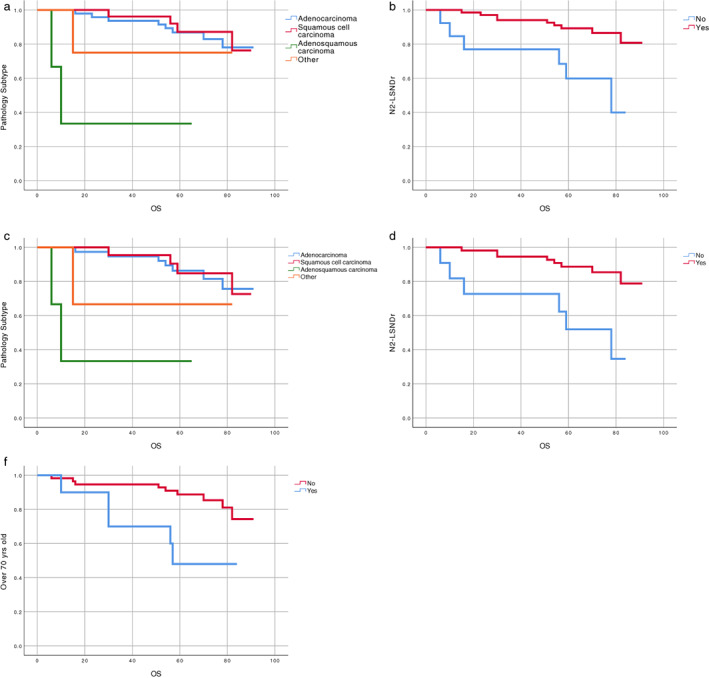
Kaplan–Meier survival curves of overall survival (OS) for all the enrolled stage IB patients: a. pathology subtype; b. LSNDr status. Kaplan–Meier survival curves of OS for stage IB patients who only received surgery: c. pathology subtype; e. LSNDr status; f. age

In univariable analysis of OS, among all the enrolled stage IB patients, those with ASC had inferior OS (univariable HR 0.46, 95% CI 0.05–4.14, *p* = 0.001) and those with N2‐LSNDr had superior OS (univariable HR 4.31, 95% CI 1.53–12.12, *p* = 0.003) (Table 2). Among stage IB patients who did not receive adjuvant chemotherapy following surgery, those with ASC had inferior OS (univariable HR 0.42, 95% CI 0.05–3.75, *p* = 0.003) and those with N2‐LSNDr had superior OS (univariable HR 4.88, 95% CI 1.69–14.10, *p* = 0.001). In addition, advanced age (≥70 years old) is also an adverse factor for OS (univariable HR 0.29, 95% CI 0.10–0.87, *p* = 0.018) (Figure 2 and Table 3).

### Multivariable of prognostic factors

In multivariable analyses, we found that among all the enrolled stage IB patients, those with *T* > 3.5 cm exhibited longer DFS (multivariable HR 5.42, 95% CI 1.19–24.68, *p* = 0.029) and those with N2‐LSNDr demonstrated longer OS (multivariable HR 3.41, 95% CI 1.03–11.45, *p* = 0.045) (Table 2). In stage IB patients who did not receive adjuvant chemotherapy following surgery, N2‐LSNDr can prolong the patient's OS (multivariable HR 4.69, 95% CI 1.33–16.54, *p* = 0.016) and advanced age is an adverse factor for OS (multivariable HR 0.24, 95% CI 0.07–0.81, *p* = 0.021) (Table 3).

In addition, this study did not find an effect of surgical methods on DFS (*p* = 0.517, *p* = 0.534) and OS (*p* = 0.735, *p* = 0.840) in all stage IB patients and those who did not receive adjuvant chemotherapy. The influence of vascular nerve invasion on DFS (*p* = 0.387, *p* = 0.540) and OS (*p* = 0.432, *p* = 0.592) was not observed in all stage IB patients and those who did not receive adjuvant chemotherapy.

## DISCUSSION

Although Sung et al. reported that lung cancer incidence has dropped to second place globally, its mortality remains the leading cause of cancer‐related deaths.^1^ NSCLC accounts for approximately 85% of lung cancers,^9^ and remarkable advances have recently been realized in surgery and adjuvant treatment for various NSCLC stages. Although some guiding recommendations for postoperative adjuvant treatment of stage IB NSCLC are included in the latest National Comprehensive Cancer Network (NCCN) guidelines, several issues remain unclear. In clinical practice, we also have some uncertainty regarding the formulation of treatment plans for stage IB NSCLC patients, and we are interested in factors that can affect the prognosis of stage IB patients with tumor diameters of between 3 and 4 cm. For these reasons, we designed this study to investigate the factors influencing the prognosis of T2aN0M0 NSCLC patients by retrospectively analyzing data from IB patients who underwent surgical treatment at our center. We focused our research on a small group, but we still obtained some clinically guiding results.

In univariate analysis, we found that ASC is an adverse factor affecting DFS and OS. ASC is defined as a subtype of NSCLC comprising adenomatous and squamous components, each accounting for at least 10%.^10^ Most ASC patients are males older than 50 years who smoke cigarettes.^11^ ACS is believed to be more aggressive than single‐histology adenocarcinoma or squamous carcinoma with poorer outcome.^12^, ^13^, ^14^ Filosso et al. stated that patients with stage I ASC undergoing radical resection had 3‐year and 5‐year survival rates close to those of patients with stage IIIA adenocarcinoma and squamous cell carcinoma.^15^ However, ASC has not been thoroughly studied yet due to its low incidence (0.6–4.2%).^14^, ^16^, ^17^, ^18^ Although ASC patients accounted for only 3.75% of all recruited patients in this study, their prognosis was worse than that of other patient subgroups. Both in all the enrolled stage IB patients and stage IB patients who underwent only surgical treatment, ASC patients had shorter DFS (*p* = 0.036, *p* = 0.037) and OS (*p* = 0.001, *p* = 0.003) compared with other pathological subtypes. At present, high‐risk factors in stage IB treatment in NCCN guidelines do not involve the pathological subtype of ASC. We believe that if more in‐depth research confirms the above findings, it may be added to high‐risk factors. From Rami‐Porta et al.’s findings according to the 8th edition TNM staging system, while tumor diameters range from 1 to 5 cm in N0M0 NSCLC patients, the prognosis gradually deteriorates for every increase of 1 cm in the tumor, and the 5‐year survival rate can decline from 92% to 47%.^5^ The patients included in this study were NSCLC patients with diameters of 3–4 cm. We divided them into two subgroups according to whether they were >3.5 cm or ＜< 3.5 cm. After analysis, we discovered that patients with *T* > 3.5 cm had a longer DFS regardless of whether all stage IB patients were enrolled (*p* = 0.021) or those who received surgery only (*p* = 0.016). In Cox regression analysis, it was also found that *T* > 3.5 cm was a favorable factor for DFS in all stage IB patients (*p* = 0.029). These findings appear to be inconsistent with our previous cognitive and clinical experiences. Further research revealed that when all stage IB patients were compared using the rank‐sum test, the number of lymph node dissection locations in the N1 group of patients with *T* > 3.5 cm was greater, exhibiting a statistical difference (*p* = 0.034). Although no statistical difference was observed between stage IB patients who did not receive adjuvant chemotherapy (*p* = 0.127), the number of lymph node dissection locations is significantly greater in the N1 group of patients with *T* > 3.5 cm (mean rank 37.50 vs. 30.90). Zhang et al. confirmed that fewer lymph node dissections are associated with a higher recurrence rate in patients with stage IB‐IIA NSCLC.^19^ Therefore, in this study, although the tumor diameter was larger in *T* > 3.5 cm patients, the difference in lymph node dissection led to DFS differences between the two subgroups, indicating that tumor diameter's impact on the prognosis of patients at this stage remains questionable.

Previous research has demonstrated that lymph node sampling or dissection is critical in precise nodal staging since it identifies lymph node involvement, ascertains the disease extent, and evaluates the therapeutic effect on lymph node metastatic lesion clearance.^20^, ^21^, ^22^ Recently, Liang et al. also found that a greater number of examined lymph nodes is associated with more accurate nodal staging and improved long‐term survival of patients with stage I–IIIA resected NSCLC.^23^ Based on IASLC recommendations, we divided enrolled patients into two groups (LSNDr and non‐LSNDr), according to the number of lymph node dissection regions. We discovered that patients receiving N2‐LSNDr had a longer DFS (*p* = 0.020, *p* = 0.005) and OS (*p* = 0.003, *p* = 0.001), regardless of whether they were all enrolled stage IB patients or stage IB patients who received only surgery, and that N2‐LSNDr could also prolong OS (*p* = 0.045, *p* = 0.016) in the multivariate analysis of the two groups. Jarabo et al. recently discovered that a high proportion of surgical procedures did not follow IASLC recommendations for lymph node surgical resections.^24^ Our findings not only confirm the importance of N2 lymph node dissection for the prognosis of early NSCLC but also imply that surgical procedures for NSCLC should be conducted following IASLC recommendations.

The age limitation for NSCLC patients has declined as surgical techniques, perioperative treatment, and concepts advance. According to some reports, over 80 years of age is now confirmed as no longer a contraindication for surgery in patients with stage I NSCLC.^25^, ^26^, ^27^ However, while age is becoming less restrictive for surgery, it clearly affects the prognosis of NSCLC patients. Previous studies have also confirmed that age is a poor prognostic factor for patients with early‐stage NSCLC.^19^, ^28^ Of the patients included in this study, 13 (16.26%) cases were ≥70 (range 70–86) years old. Only two patients (both<75 years old) received adjuvant chemotherapy for vascular invasion due to their advanced age. Among stage IB patients who did not receive adjuvant chemotherapy, older patients had shorter DFS (*p* = 0.049) and OS (*p* = 0.018), and in Cox regression analysis older age is also an adverse factor for OS (*p* = 0.021).

Sublobectomy was previously considered to be a significant predictor of poor prognosis in patients with stage IB NSCLC,^28^ and vessel invasion is also a high‐risk factor for recurrence in stage I patients.^29^ However, in this study we found no impact of the above two factors on the prognosis of all patients who were enrolled in stage IB and those who did not receive adjuvant chemotherapy following surgery. This is possibly due to the relatively small number of patients enrolled and the relatively low proportion of the above two types of patients (5%, 3.75%).

This study exhibits some limitations. First, like most studies on stage IB NSCLC, it is also a retrospective study. Unavoidable selection bias will exist. Second, since the enrolled patients underwent treatment between 2013 and 2016, no targeted therapy and immunotherapy exist in the postoperative adjuvant treatment regimens, and some patients have particular concerns regarding postoperative adjuvant chemotherapy due to its side effects. Consequently, the proportion of patients in this group receiving adjuvant chemotherapy after surgery is relatively low (17.5%). Moreover, this study is a single‐center study with a relatively small number of cases recruited in the group. Accordingly, no additional analysis was conducted on patients receiving postoperative adjuvant chemotherapy in the data analysis. Through retrospective analysis of data from T2aN0M0 (stage IB) NSCLC patients surgically treated at our center, this study found that N2‐LSNDr were independent influencing factors affecting the prognosis of all included stage IB patients and stage IB patients who underwent only surgery. ASC was associated with worse prognosis of T2aN0M0 NSCLC. Older age could shorten the DFS and OS of stage IB patients without adjuvant chemotherapy. The tumor diameter's impact on the prognosis in stage IB patients remains debatable. Due to the limited sample size, this study demonstrated that surgical methods and vascular nerve invasion had no impact on prognosis. We anticipate future multicenter prospective studies on surgical and postoperative adjuvant treatments of NSCLC patients with stage T2aN0M0 (stage IB).

## CONFLICT OF INTEREST

The authors declare that they have no competing interests.
